# Idiosyncratic nature of lactation reveals link to breast cancer risk

**DOI:** 10.21203/rs.3.rs-4601714/v1

**Published:** 2024-06-28

**Authors:** Mrittika Chattopadhyay, Edmund Charles Jenkins, William Janssen, Thelma Mashaka, Doris Germain

**Affiliations:** 1- Icahn School of Medicine at Mount Sinai, Tisch Cancer Institute, Department of Medicine, Division of Hematology/ Oncology, New York, 10029, NY, USA.; 2- Icahn School of Medicine at Mount Sinai, Microscopy and Advanced Bioimaging Core, New York, 10029, NY, USA.

## Abstract

Breastfeeding protects against breast cancer in some women but not others, however the mechanism remains elusive. Lactation requires intense secretory activity of the endoplasmic reticulum (ER) for the production of milk proteins and ER-mitochondria contacts for lipid synthesis. We show that in female mice that share the same nuclear genome (BL/6) but differ in mitochondrial genomes (^C57^ or ^NZB^), the biological processes engaged during lactation are entirely different at the sub-cellular organization and transcriptional levels resulting in anti-tumorigenic lactation in BL/6^C57^ females and pro-tumorigenic lactation in BL/6^NZB^ females. Single cell sequencing identified a sub-population of cells, uniquely amplified during lactation in BL/6^NZB^ females, which shares the genetic signature that characterizes post-partum breast cancer (PPBC) in humans relative to matched breast cancers in never pregnant women. Our data indicate that differences in ER and mitochondrial-stress responses during lactation between genotypes inadvertently leads to loss of p53 tumor suppressor function in BL/6^NZB^ females allowing the expansion of the PPBC-like sub-population of cells. Overall, our data reveals the unexpected idiosyncratic nature of lactation and its impacts on the risk of the development of PPBC.

While pregnancy at an early age is associated with a reduction in the overall life-time risk of developing breast cancer^1^, pregnancy after the age of 30 is associated with increased risk of breast cancer with the risk peaking 6 years post-partum but remaining higher with increasing age of the mother over decades^1^. These post-partum breast cancers are more aggressive and metastatic^2–5^. Since in developed countries, women tend to have their children after the age of 30, pregnancy represents an important etiological factor in breast cancer today. One major breakthrough regarding increased risk of breast cancer following pregnancy is the discovery that post-pregnancy involution is tumorigenic^6–12^.

However, a large study involving over 50, 000 women with breast cancer and nearly 100, 000 without breast cancer, concluded that extended breastfeeding is protective against breast cancer^13^. The simple fact that such a large sample size was required to reach this conclusion reflects the fact that breastfeeding involves multiple variables including genetic, environmental and dietary factors and suggests that lactation is in fact not protective for some women. In agreement with this possibility, one study indicated that excessive breastfeeding may promote breast cancer^14^.

At the sub-cellular level, lactation implicates the secretion of proteins and lipids, both of which involve the endoplasmic reticulum (ER). Accumulation of proteins or lipids in the ER activates a stress response referred as ER-stress, which is generally adaptive and restores homeostasis of the ER. However, under conditions where stress in the ER cannot be resolved, the ER-stress response results in apoptosis^15^.

The ER is in close physical contact with the mitochondria and these contacts between both organelles regulate mitochondrial fission^16,17^, affect the function of both organelles including lipid metabolism^18,19^ and were reported to be modulated during lactation^20^. Stress in the mitochondria also activates a stress response^21–24^.

We chose to investigate the impact of lactation in mice that share the same nuclear genome in the C57BL/6 genetic background (BL/6) but differ in mitochondria genomes, either from the C57BL/6 or NZB/OlaHsd (^C57^ or ^NZB^) background^25^. Since the number of nucleotide differences in the mitochondrial DNA between NZB/OlaHsd and BL/6C57 mice is within the same range as that observed between the European and African DNA haplotypes in the human population, these mice represent a model that reflects the natural diversity in mitochondrial genetics in humans^25^.

Our results indicate that mitochondrial haplotypes have a profound impact on the fundamental biology of lactation, resulting in pro-versus anti-tumorigenic environment. Notably in the setting of a pro-tumorigenic lactation, escape from ER-stress response inadvertently represses the expression of the tumor suppressor p53 allowing the expansion of a sub-population of cells that shares the genetic profile that characterizes post-partum breast cancers (PPBC) relative to matched breast cancers in never pregnant women (NPBC) 26. Our findings indicate that in the subset of women carrying mitochondrial haplotypes where the expansion of the PPBC-like cell population during lactation is observed, the susceptibility to develop PPBC is increased. We also show that dietary supplement targeting ER-stress can convert pro-tumorigenic lactation into a protective lactation, therefore providing an actionable intervention to expand the beneficial effect of breastfeeding to more women.

## Results

### Lactation engages drastically different biology in female mice that differ only in mitochondria.

To gain insights into the impact of different mitochondria on lactation, we harvested the mammary glands over a time course including day 8, 14 and 21 of lactation in BL/6^C57^ and BL/6^NZB^ female mice and performed bulk RNAseq.

**Figure 1.**
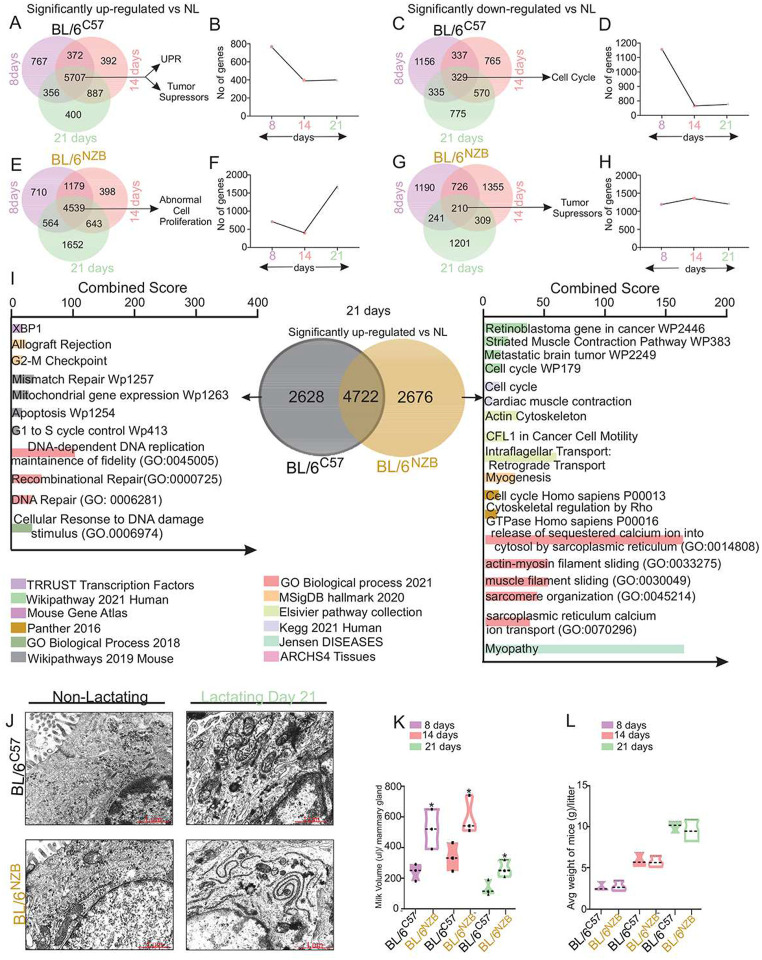
Differential regulation of cell cycle and tumor suppressors pathways at day 21 of lactation. **A–H**, RNA seq analysis was performed on non-lactating (NL) and lactating (L) BL/6^C57^ or BL/6^NZB^ female mice on day 8, 14 and 21 of lactation. Venn diagram indicating the number of genes significantly (adjusted P < .05) upregulated or downregulated either in BL/6^C57^ or BL/6^NZB^ female mice at day 8, 14, 21 of lactation compared to their non lactating (NL) controls (n= 3) (A, C, E, G). Graphs showing the trend of genes exclusively upregulated or downregulated at day 8, 14 and 21 in BL/6^C57^ or BL/6^NZB^ mice (B, D, F, H). **I**, Venn diagram indicating the number of genes significantly (adjusted P < .05) in BL/6^C57^ mice or BL/6^NZB^ mice at day 21 of lactation compared to their non lactating (NL) control (n = 3) (middle). The genes exclusively upregulated in BL/6^C57^ or BL/6^NZB^ mice were subjected to Enrichr and are represented in the graphs according to their combined score. All pathways shown are statistically significant. **J**, Electron Microscopy was performed on the mammary glands of BL/6^C57^ or BL/6^NZB^ mice at day 21 of lactation compared to their non lactating (NL) control (n = 3). **K**, Graph of milk volumes per gland collected from either BL/6^C57^ or BL/6^NZB^ mice on day 8, 14 and 21 of lactation. Student’s t test, two-tailed, *p < 0.05, **p < 0.01, ***p < 0.001; mean ± SD; n = 6 mice/group. **L**, Graph showing average weight of mice per litter at day 8, 14, 21 of lactation. Student’s t test, two-tailed; mean ± SD; n = 6 mice/group.

This analysis revealed that 767 genes were specifically up-regulated at day 8, 392 at day 14 and 400 at day 21 of lactation in BL/6^C57^ females, indicating that the number of genes specifically expressed in early, mid and late lactation decreases over time (Fig. 1A–B). The same pattern of decreased gene transcription over time during lactation was observed for the genes that are down-regulated (Fig. 1C–D). We then focused on the genes that are commonly up-regulated or down-regulated at all time points of lactation and found 5707 common genes that are up-regulated (Fig. 1A) and 329 common genes that are down-regulated (Fig. 1C). The pathway analysis of the 5707 genes commonly up-regulated in BL/6^C57^ females revealed pathways related to the UPR and tumor suppressors, notably p53 (Fig. 1A, Suppl. Fig. 1A), while cell cycle was the main pathway observed from analysis of the 329 common genes that are down-regulated (Fig. 1C, Suppl. Fig. 1A).

The same analysis was performed in the BL/6^NZB^ females. We found that in contrast to the BL/6^C57^, the number of genes specifically up-regulated at day 8, 14 and 21 increased through the time course of lactation (Fig. 1E–F), while the genes that are down-regulated at specific days of lactation remained relatively constant (Fig. 1G–H). This global analysis indicates that the overall patterns of gene expression during lactation differ widely between genotypes. Focusing on the genes that are common between lactation time points in the BL/6^NZB^ females, 4539 were found to be up-regulated and associated with abnormal proliferation (Fig. 1E, Suppl. Fig. 1B), while 210 genes were commonly down-regulated and linked to cell death and the p53 tumor suppressor (Fig. 1G, Suppl. Fig. 1B).

We then compared the genes that are uniquely up-regulated across lactation time points but that are specific to each genotype. We found that 4722 genes were common between the genotypes but 2628 and 2676 were unique to the BL/6^C57^ and BL/6^NZB^ females respectively (Fig. 1I). Pathways analysis of the 2628 genes up-regulated specifically in the BL/6^C57^ females highlighted ER-stress, apoptosis, cell cycle checkpoints (Fig. 1I). In contrast, the 2676 genes specifically up-regulated in the BL/6^NZB^ females revealed a cell cycle signature as well as a muscle related signature (Fig. 1I).

These analyses revealed that 1) the global number of genes over the period of lactation varies between genotypes and 2) the pathways activated during lactation also differ widely, therefore suggesting an entirely different biology is taking place during lactation between genotypes. To gain further insight into these differences at the cellular level, we performed electron microscopy. This analysis indicated that the number and morphology of the endoplasmic reticulum and mitochondria are widely different between genotypes at the cellular level, notably a dilated ER network is observed in BL/6^C57^ females, while a thin, elongated and curvy ER network characterizes the secretory cells during lactation in BL/6^NZB^ females (Fig. 1J). To test if these differences affect the secretion of milk, we measured the volume of milk and found that both genotypes produce milk, with the BL/6^NZB^ showing increased secretion (Fig. 1K). Additionally, no difference in the weight of pups over time was observed (Fig. 1L) suggesting that the nutritional value of milk between genotypes is also similar. Collectively therefore, despite the fact that in females from both genotypes, lactation fulfills its physiological function and sustain the growth of their pups, the cellular biology and pathways activated during lactation are drastically different.

**Figure 2.**
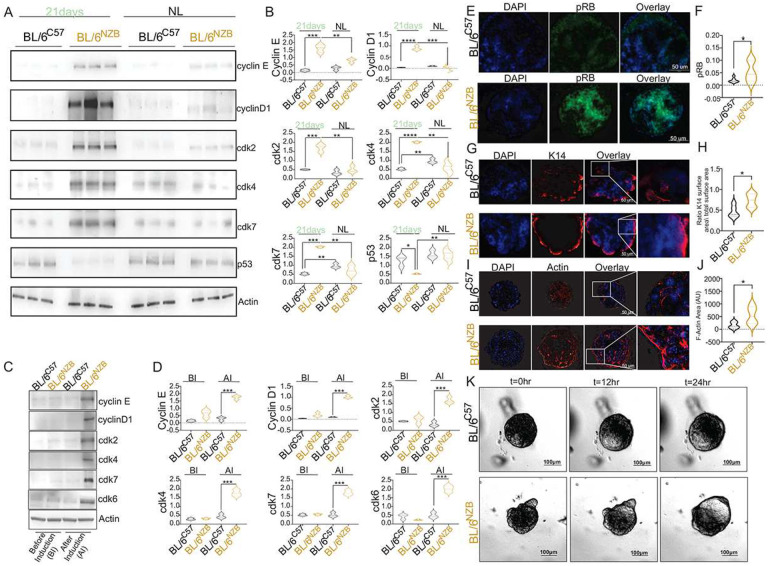
Validation of pathways identified by RNAseq *in vivo* and *ex vivo*. **A–B**, Western blot analysis of indicated cell cycle markers and p53 in non lactating (NL) and lactating (Day 21) in mammary glands from BL/6^C57^ or BL/6^NZB^ female mice (A). Graphs of quantification of western blot analysis (B). Student’s t test, two-tailed, *p < 0.05, **p < 0.01, ***p < 0.001; mean ± SD; n = 3 mice/group. **C,** Western Blot analysis of cell cycle markers in mammary organoids derived from BL/6^C57^ or BL/6^NZB^ female mice before (BI) or after induction (AI) with 1μg/ml prolactin, 1μg/ml hydrocortisone and 40 μg/ml oxytocin on the 6th day of organoid culture. **D**. Graphs showing the quantification of western blot analysis in C. Student’s t test, two-tailed, *p < 0.05, **p < 0.01, ***p < 0.001; mean ± SD; n = 3 (D). **E–J**, Mammary organoids from BL/6^C57^ or BL/6^NZB^ mice induced with 1μg/ml prolactin, 1μg/ml hydrocortisone and 40 μg/ml oxytocin were fixed and stained for pRB, K-14, Actin and Dapi. (E, G, I) Representative image of pRB, K-14, Actin, Dapi and overlay. The scale bar represents 50 μm. (F, H, J) Graph showing pRB stained region, K-14 surface area compared to total surface area, Actin-stained area which were analyzed using image J software. Student’s t test, two-tailed, *p < 0.05; mean ± SD; n = 3. **K**, Primary mammary organoids isolated from non-lactating BL/6^C57^ or BL/6^NZB^ mice were grown for 6 days before induction with 1μg/ml prolactin and 40 μg/ml oxytocin. Images show representative organoids at the indicated time. The scale bar represents 100 μm.

In order to validate the data obtained by RNAseq, we first analyzed the specific cell cycle genes that led to the identification of this pathway and analyze mammary glands at day 21 of lactation by Western blotting. We found that all 5 cell cycle markers tested (cyclin E, D1, cdk2, 4 and 7) are significantly higher in BL/6^NZB^ females, not only relative to the BL/6^C57^ females at day 21 of lactation, but also to BL/6^NZB^ females that are not lactating (Fig. 2A–B). Additionally, since a tumor suppressor gene signature driven by p53 was identified as down regulated specifically in the BL/6^NZB^ (Fig. 1), we also analyzed p53 level and confirmed it is down-regulated specifically during lactation in the BL/6^NZB^ females (Fig. 2A, B).

To further validate the results, we created 3D lactation organoids using primary mammary glands from virgin females from both genotypes. In this system, organoids are first allowed to get established in culture and are then induced to mimic lactation by stimulation with prolactin and oxytocin. The organoids were harvested after 24 hours of stimulation for immunofluorescence and Western analysis of the same cell cycle markers. Western analysis of cell cycle markers showed that all markers are specifically up-regulated in the BL/6^NZB^ organoids following induction with prolactin and oxytocin (Fig. 2C, D). Additionally, since the retinoblastoma was specifically up-regulated in the BL/6^NZB^ females (Fig. 1), we analyzed activation of Rb using immunofluorescence of phosphorylated Rb and confirmed it is up-regulated in BL/6^NZB^ lactation organoids specifically (Fig. 2E, F).

To validate the muscle contraction and cytoskeleton signatures observed in the RNAseq data (Fig. 1), we analyzed myoepithelial cells using K14 and actin and found that in both cases, the staining is higher in the organoids of BL/6^NZB^ females (Fig. 2G–J). Finally, to assess if this phenotype has any functional consequence on the production of milk, we performed live microscopy of the organoids over a time course following addition of prolactin. We found that consistent with the increased milk production found *in vivo* (Fig. 1K), the filling of the lumen of the BL/6^NZB^ organoids is visibly more pronounced after addition of prolactin (Fig. 2K). Collectively, these results confirmed that the pathways detected by RNAseq can be observed *in vivo* and *ex vivo* in lactation organoids.

### Lactation leads to an anti-versus pro-tumorigenic microenvironment in the BL/6^C57^ and BL/6^NZB^ females respectively.

**Figure 3.**
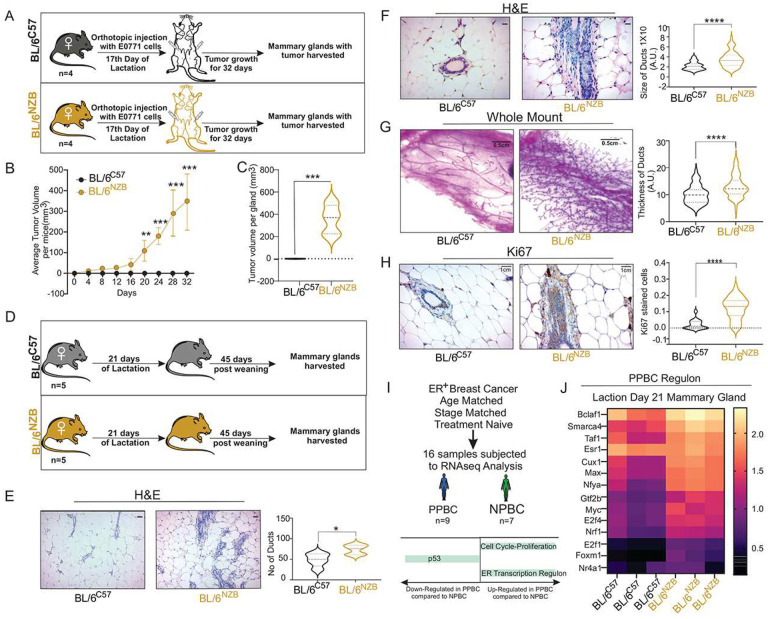
Post-partum tumor formation and mammary hyperplasia is observed in BL/6^NZB^ female mice. **A**, Diagram of the experimental design in indicated female mice (n = 4). **B–C**, 1X10^5^ E0771 cells were injected in inguinal mammary glands of BL/6^C57^ or BL/6^NZB^ mice on day 17 of lactation. Graph depicts the mean tumor volume over time (B). Quantification of tumor volumes at day 32 (C). Quantification at each time point represents the average of 3 BL/6^C57^ mice relative to the average value of 3 BL/6^NZB^ mice. Student’s t test, two-tailed, *p < 0.05, **p < 0.01, ***p < 0.001; mean ± SD; n = 4 mice/group. **D**, Diagram of the experimental design in indicated genotypes (n= 4 female mice). **E–F**, H&E section of mammary glands from BL/6^C57^ or BL/6^NZB^ mice harvested after 45 days post completion of 21 days of lactation. Scale bar represents 1 cm. Graphs showing number of ducts or size of ducts analyzed using image J software. Student’s t test, two-tailed, *p < 0.05, ****p < 0.0001; mean ± SD; n = 5 mice/group. **G**, Representative whole mount of mammary gland from BL/6^C57^ or BL/6^NZB^ mice harvested after 45 days post-partum after completion of 21 days of lactation. The scale bar represents 0.5 cm. Graph showing thickness of ducts were analyzed using image J software. Student’s t test, two-tailed, ****p < 0.0001; mean ± SD; n = 5 mice/group. **H**, Representative image of IHC of Ki67 in mammary glands derived from BL/6^C57^ or BL/6^NZB^ mice harvested after 45 days post-partum after completion of 21 days of lactation. The scale bar represents 1 cm. Graph showing number of Ki67 stained cells were analyzed using image J software. Student’s t test, two-tailed, ****p < 0.0001; mean ± SD; n = 5 mice/group. **I**, Schematic of patient’s selection and pathways identified in the human study comparing post-partum breast cancer (PPBC) to never pregnant breast cancer (NPBC). **J**, Heatmap of the 14 transcription factors of the post-partum breast cancer (PPBC) regulon at day 21 of lactation in BL/6^C57^ and BL/6^NZB^ female mice.

Since increased proliferation and inhibition of p53 are hallmark of cancer, we next investigate whether the differential activation of these pathways correlates with post-partum proliferation status. To test if lactation impacts the growth of cancer cells, lactating females of both genotypes were injected with the EO771 mouse mammary tumor cells at day 17 of lactation and the formation of tumor tested 45 days after (Fig. 3A). Remarkably, while tumors grew rapidly in the BL/6^NZB^ females, none of the BL/6^C57^ females develop tumors (Fig. 3B, C). As the EO771 model is known to be an aggressive model of breast cancer, this result indicates that the microenvironment of lactating mammary gland in the BL/6^C57^ females is dominant over these aggressive cancer cells and demonstrate a stunning resilience against cancer formation in these mice.

Additionally, since the RNAseq was performed on the mammary gland, we also tested if these pathways affect the growth of endogenous epithelial cells of the mammary gland. Therefore, we allowed females from both genotypes to lactate for 21 days, separated the mothers from their pups and harvested the mammary glands of the mothers 45 days after weaning (Fig. 3D). We then performed three complementary analyses; 1) histology by H&E staining, 2) whole mount analysis and, 3) staining for Ki67, as a marker of proliferation. We found that the number (Fig. 3E) and size (Fig. 3F) of the ducts are smaller in the BL/6^C57^ females in 45 days post-partum mammary glands compared to BL/6^NZB^ females. Most strikingly, whole mount analysis revealed that while the mammary glands of BL/6^C57^ females resemble that of virgin mice, this analysis showed significant proliferation in the mammary gland at day 45 post-lactation in the BL/6^NZB^ females (Fig. 3G) and the increased proliferation was confirmed using ki67 staining (Fig. 3H).

We concluded that mitochondrial genetics alone is able dictate the physiological response to lactation, where lactation promotes the formation of an anti-tumorigenic post-partum mammary gland in the BL/6^C57^ females. However, a pro-tumorigenic environment is observed after lactation in the BL/6^NZB^ females raising the possibility that BL/6^NZB^ females may represent a model of susceptibility to the development of post-partum breast cancers (PPBC).

Recently, a genetic signature the distinguish PPBC from matched breast cancer in never pregnant women was identified^26^. In this study, the cohort consisted of 16 estrogen receptor alpha (ERα) positive breast cancer patients that were matched in term of age, tumor grade and treatment and were divided into 9 patients with an history of pregnancy and 7 patients who never had children (Fig. 3I). RNAseq analyses of these tumors revealed a PPBC profile consisting of high cell cycle score, loss of p53 and elevated expression of a transcriptional regulon containing 14 transcription factors^26^. Since we already found elevated cell cycle markers and low p53 expression in BL/6^NZB^ females during lactation (Figs. 1, 2), to further explore the similarity between PPBC in humans and the mammary glands of BL/6^NZB^ lactating females, we investigated the expression of individual transcription factors that defined the PPBC-regulon in humans in BL/6^NZB^ lactating females compared to BL/6^C57^ females. We found that the PPBC transcriptional regulon is enriched in BL/6^NZB^ lactating females compared to BL/6^C57^ females (Fig. 3J). These results therefore demonstrate that all aspects of the PPBC signature (cell cycle, p53 and regulon) are recapitulated in BL/6^NZB^ females during lactation.

### Single cell RNAseq identifies a high-cell cycle score and high-regulon positive sub-population that is uniquely expands during lactation in BL/6^NZB^ females.

Based on the findings of the overlap between the PPBC signature and lactation in BL/6^NZB^ females, we hypothesized that a cell population may arise during lactation in these female mice and that this cell population maybe more prone to transformation upon acquisition of an oncogenic mutation such that it is primed to develop into post-partum breast cancers. To test this hypothesis, we performed single cell RNAseq on mammary glands of 3 non-lactating and 3 lactating females (day 21 of lactation) from both genotypes.

**Figure 4.**
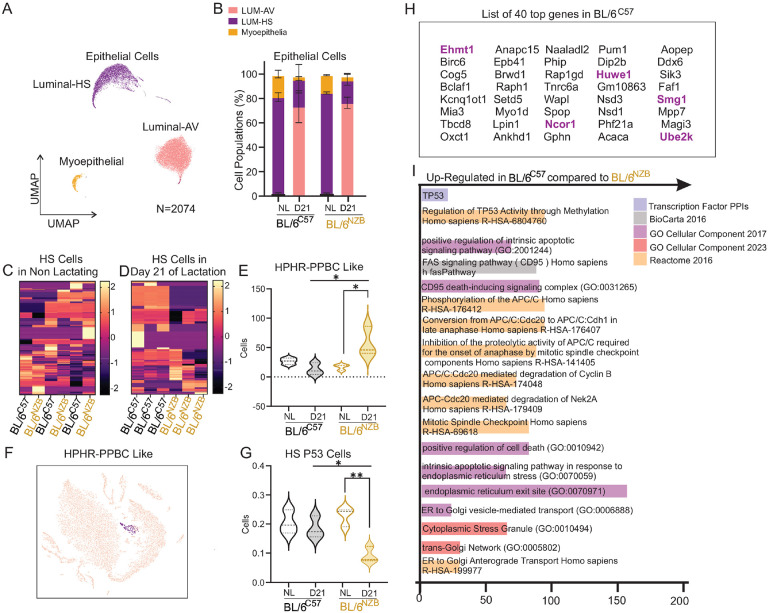
Identification of PPBC-like sub-population of cells during lactation in BL/6^NZB^ female mice. **A,** UMAP plot of Luminal Alveolar (Luminal AV), Hormone sensing (HS) and myoepithelial cell clusters identified by expression of characteristic markers. **B,** Relative proportion of epithelial cell types per genotype in non-lactating (NL) and day 21 lactating (D21) females. **C,** Hierarchical clustering heatmaps of differential gene expression of luminal HS cells in virgin BL/6^C57^ and BL/6^NZB^ females. **D**, Hierarchical clustering heatmaps of differential gene expression of luminal HS cells in lactating BL/6^C57^ and BL/6^NZB^ females **E**, Number of PPBC-like cells (defined by high cell cycle score and high regulon positivity, see text) in non-lactating (NL) and lactating day 21 (D21) BL/6^C57^ and BL/6^NZB^ females. **F**, Representative image of PPBC-like positive cell cluster. **G**, Number of luminal HS cells expressing p53 in non-lactating (NL) and lactating day 21 (D21) BL/6^C57^ and BL/6^NZB^ females. **H**, List of the top 40 differentially up-regulated genes in luminal HS cells in BL/6^C57^ relative to BL/6^NZB^ female mice at day 21 of lactation. Bold indicated p53 transcriptional targets. **I**, Pathways related to the expression of the 40 genes shown in H. Databases where these pathways were identified are color coded.

Since our goal was the identification of a potential cell population at the origin of PPBC, we focused on the epithelial compartment, which is composed on three main cell types, the luminal alveolar cells (AV) the luminal hormone sensing cells (HS) and the myoepithelial cells (ME) (Fig. 4A). The relative distribution of these sub-types of cells confirmed an expansion of the alveolar cells during lactation (Fig. 4B) but no significant differences were observed between genotypes (Fig. 4B). Since the PPBC signature in humans was identified in ERa positive breast cancers, we analyzed the luminal HS cellular sub-type since they are characterized by expression of the ERa. We found that in virgin females, the luminal HS cells did not significantly differ in gene expression resulting in no hierarchical clustering between genotypes (Fig. 4C). The transcriptional profiles of luminal HS cells during lactation however, resulted in a clear hierarchical clustering by genotypes (Fig. 4D) indicating that lactation differentially modulates gene expression in these cells in BL/6^C57^ and BL/6^NZB^ females.

To test our hypothesis that a PPBC-like population of cells may be present in the BL/6^NZB^ females, we then interrogate our data set to identify the number of cells that express both a high cell cycle score, as defined in the human study, and are also positive for the transcription PPBC-regulon^26^. Therefore, we define PPBC-like cells as those with high cell cycle score and high PPBC-regulon. We found that while such cells were identified in all conditions, the number of PPBC-like cells is significantly higher in BL/6^NZB^ during lactation (Fig. 4E–F). Further, we found that 61% of PPBC-like cells also express the luminal HS markers.

The observation that PPBC-like cells are elevated specifically during lactation in BL/6^NZB^ females indicate that an event that is unique to these females during lactation allows their proliferation. As we already identified that loss of p53 expression is specific to the lactating BL/6^NZB^ females (Figs. 1, 2) and loss of p53 characterized PPBC in humans^26^, we next interrogate our data set for the number of luminal HS cells that are positive for p53. We found that the number of p53 positive luminal HS cells is significantly lower in the BL/6^NZB^ females relative to lactating BL/6^C57^ or virgin females from both genotypes (Fig. 4G).

Having established that the gene expression profiles of luminal HS cells differ between genotypes during lactation (Fig. 4D), to independently assess the role of p53, we selected the top 40 genes (*p*< 0.0002) (Fig. 4H) that show the most highly differential expression between luminal HS cells from BL/6^C57^ and BL/6^NZB^ females during lactation for pathway analysis. These 40 genes were up-regulated in BL/6^C57^ lactating females relative to BL/6^NZB^ lactating females. This analysis revealed that p53 is among the top transcription factor (Fig. 4I) and pathways related to ER-stress and apoptosis are elevated in in luminal HS cells in BL/6^C57^ females during lactation.

Collectively, these results support the conclusion that a PPBC-like sub-population of cells are allowed to expand during lactation, specifically in BL/6^NZB^ females and that this expansion correlates with lack of expression of p53.

### Idiosyncrasy of lactation leads to differential activation of organellar stress responses.

While the regulation of the tumor suppressor p53 under stress conditions such as irradiation, has been extensively studied, the regulation of p53 under physiological settings has also been well described^27–29^. Notably, ER-stress and metabolism have been shown to affect p53^27,29–33^. Considering that we observed loss of p53 expression during lactation in the BL/6^NZB^ females (Fig. 2A) and lactation engages robust secretion that alters both ER-stress and mitochondria^34,35^, we tested whether the idiosyncrasies of lactation between genotypes also results in differential activation of stress pathways in the ER and mitochondria.

**Figure 5.**
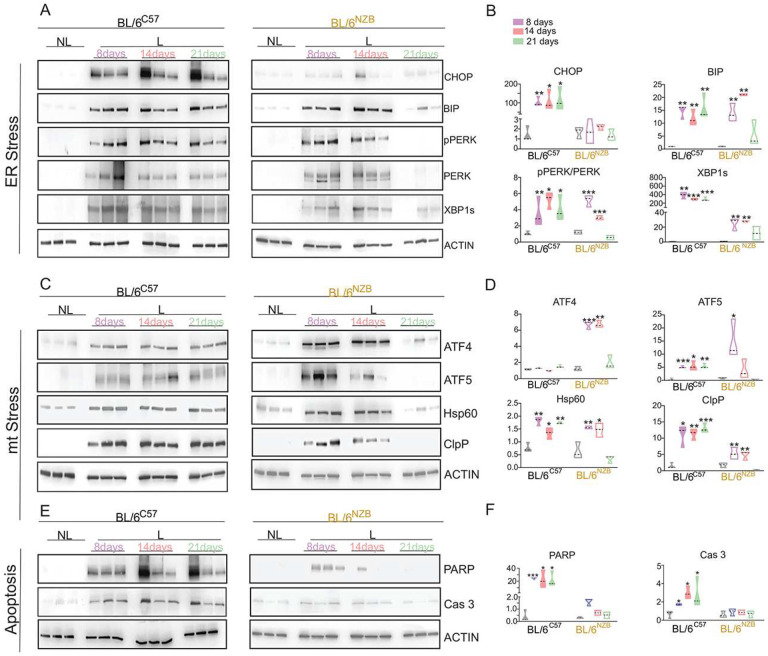
Differential activation of ER and mitochondrial stress pathways during lactation in BL/6^C57^ and BL/6^NZB^ females. Western blot analysis of mammary glands from non-lactating (NL) and lactating (L) at day 8, 14 and 21 in BL/6^C57^ (Left) or BL/6^NZB^ (right) female mice for the indicated markers of ER-stress (A),mitochondrial-stress (C) and apoptosis (E). Westerns show the result of 3 individual BL/6^C57^ or BL/6^NZB^ mice at each time point compared to their non lactating counterpart. Quantification of ER-stress markers (**B**), mitochondrial-stress markers (**D**) and apoptosis markers (**F**). Quantification represents the expression of the average of the 3 BL/6^C57^ or 3 BL/6^NZB^ mice at day 8, 14 and 21 relatives to the average value of non-lactating (NL) 3 BL/6^C57^ or 3 BL/6^NZB^ mice, which was adjusted to 1. Student’s t test, two-tailed, *p < 0.05, **p < 0.01, ***p < 0.001; mean ± SD; n = 3 mice/group.

We performed a time course of lactation and collected mammary glands at day, 8, 14 and 21 of lactation in both BL/6^C57^ and BL/6^NZB^ females. We used CHOP, BIP, PERK, phospho-PERK and XBP-1s as surrogate markers of ER stress. We found that relative to not lactating (NL) mammary glands, the levels of all markers of the UPR^ER^ tested were significantly elevated at day 8 of lactation in both genotypes except CHOP in the BL/6^NZB^ females (Fig. 5A–B). Further, while the activation of the UPR^ER^ remained constant throughout the time course in the BL/6^C57^ females (Fig. 5A–B), an abrupt cessation of the pathway was observed at day 21 of lactation in the BL/6^NZB^ mice (Fig. 5A–B). To directly compare the level of activation between genotypes, samples isolated on the same day of lactation in both BL/6^C57^ and BL/6^NZB^ were run side by side on the same gel. This analysis confirmed that the activation of CHOP is lower in BL/6^NZB^ relative to BL/6^C57^ females at all time points. Further, while all markers remain elevated at day 21 of lactation in BL/6^C57^ mice, they were all significantly reduced at day 21 in the BL/6^NZB^ females (Suppl Fig. 2A–B). We concluded that overall lactation activates the UPR^ER^ in both strains, however, the activation is sustained in BL/6^C57^, while it is abolished at day 21 in the BL/6^NZB^ females.

Since the ER is close contact with the mitochondria, we also analyzed mitochondrial stress response (UPR^mt^) over the same time course of lactation. For the UPR^mt^, we used ATF4, ATF5, Hsp60 and ClpP as surrogate markers of the pathway and found that as observed for the ER-stress, the UPR^mt^ is also activated at day 8 of lactation relative to the non-lactating mammary gland (Fig. 5C–D) and while no difference in the activation was observed between genotypes at day 8 and 14 of lactation, at day 21 all markers were drastically decreased in the BL/6^NZB^ females (Fig. 5C–D). Since the same pattern of abrupt cessation of UPR signaling is observed for both the ER and the mitochondria, we reasoned that one trivial explanation could be that the BL/6^NZB^ females either stop lactating early or the volume of milk they produce drastically reduces between day 14 and 21 of lactation. However, we found that the volume of milk produced by the BL/6^NZB^ females is in fact larger than the BL/6^C57^ females at all time points, a point that will be discussed in detailed (Fig. 1K). This observation rules out that the cessation of UPRs signaling at day 21 in the BL/6^NZB^ females is due to the cessation of lactation. Alternatively, we reasoned that since we observed that BL/6^NZB^ females have more pups per litter than BL/6^C57^ females, another explanation for these differences maybe the number of pups between genotypes. To test this possibility, we adjusted the number of pups of BL/6^NZB^ females to match the number of pups in the BL/6^C57^ females and compare the markers of the UPRs. We found no differences in the levels of the markers (Suppl. Fig. 3) Therefore, we concluded that stress responses in the ER and mitochondria are activated by lactation but the activation is sustained in the BL/6^C57^ females while it is transient and abruptly abolished at day 21 of lactation in the BL/6^NZB^ females.

Since the induction of sustained ER stress leads to apoptosis^36–39^, we also monitored markers of apoptosis. We found that apoptosis is activated in BL/6^C57^ females exclusively (Fig. 5E–F).

Collectively, these results suggest that the opposite regulation of p53 between BL/6^C57^ and BL/6^NZB^ females under the normal physiological condition of lactation may result from their differential activation of stress pathways in the ER and mitochondria.

### Treatment with the ER-stress mediated apoptosis inducer γ-Tocotrienol, reverses the tumorigenic effect of lactation in BL/6^NZB^ females.

Our results suggest a possible link between sustained activation of ER-stress in BL/6^C57^ females, apoptosis, p53 and repression of the PPBC signature. This observation raises the possibility that an intervention aimed at sustaining the activation of ER-stress leading to activation of p53 and apoptosis, would reverse the pro-tumorigenic effect of lactation in BL/6^NZB^ females. To test this possibility, we treated BL/6^NZB^ females with γ-Tocotrienol, a natural product that is reported to induced ER-stress mediated apoptosis^40–42^.

**Figure 6.**
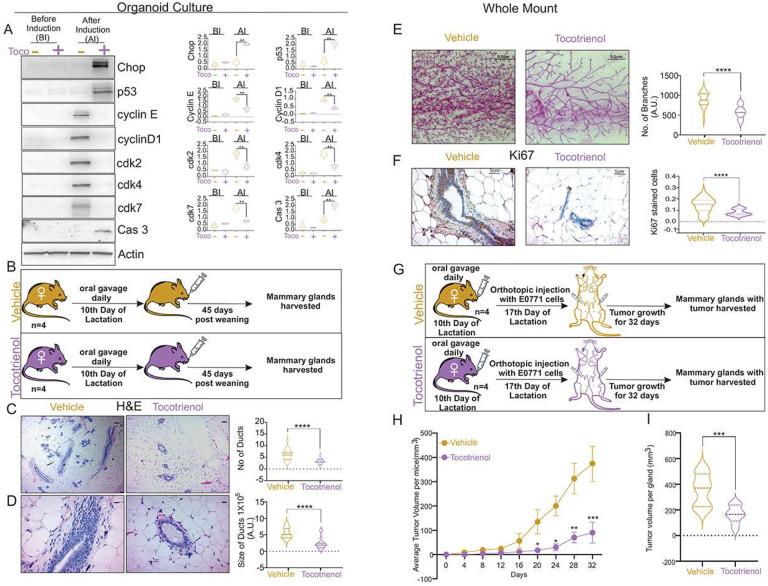
δ-Tocotrienol prevents mammary hyperplasia and tumor formation in BL/6^NZB^ mice. **A**, Organoids of mammary glands from non-lactating BL/6^NZB^ mice were treated with 20 μg/ml δ-tocotrienol starting from the 4^th^ day followed by induction with 1μg/ml prolactin, 1μg/ml hydrocortisone and 40 μg/ml oxytocin on the 6^th^ day. 22hr post induction, the organoids were subjected to western blot analysis for the indicated markers. Graphs showing the quantification of western blot analysis. Student’s t test, two-tailed, **p < 0.01; mean ± SD; n = 3. **B**, Schematic representation of experimental design. **C–D**, H&E section of mammary glands from BL/6^NZB^ mice collected 45 days post completion of 21 days of lactation treated with either vehicle (olive oil) or δ-tocotrienol (200 mg/kg body weight in olive oil, twice daily) by oral gavage starting from 10^th^ day of lactation for 4 weeks. Scale bar represents 1 cm. Graphs showing number of ducts or size of ducts analyzed using image J software. Student’s t test, two-tailed, ****p < 0.0001; mean ± SD; n = 5 mice/group. **E**, Representative whole mount of mammary gland from BL/6^NZB^ mice collected 45 days post completion of 21 days of lactation treated with either vehicle (olive oil) or δ-tocotrienol (200 mg/kg body weight. in olive oil, twice daily) by oral gavage starting from 10^th^ day of lactation for 4 weeks. The scale bar represents 0.5 cm. Graph showing thickness of ducts were analyzed using image J software. Student’s t test, two-tailed, ****p < 0.0001; mean ± SD; n = 5 mice/group. **F**, Representative image of IHC of Ki67 in mammary glands derived from above mentioned mice. The scale bar represents 1 cm. Graph showing number of Ki67 stained cells were analyzed using image J software. Student’s t test, two-tailed, ****p < 0.0001; mean ± SD; n = 5 mice/group. **G**, Diagram of the experimental design in each group, n = 4 female mice. **H–I**, BL/6^NZB^ mice were treated with vehicle (olive oil) or δ-tocotrienol (200 mg/kg body weight in olive oil, twice daily) by oral gavage starting from 10^th^ day of lactation and 1×10^5^ E0771 cells were injected in inguinal mammary glands of mice on day 17 of lactation. The mice were harvested 32 days post-partum after completion of 21 days of lactation. **H**. Graph of mean tumor volumes per mice at the given time points. Quantification at each time point represents the average of 3 BL/6^NZB^ mice treated with vehicle relative to the average value of 3 BL/6^NZB^ mice treated with δ-tocotrienol. Student’s t test, two-tailed, *p < 0.05, **p < 0.01, ***p < 0.001; mean ± SD; n = 4 mice/group. **I**, Graph of tumor volumes per gland on the day of sacrifice (day 32 post implantation).

Since we found that lactation organoids recapitulate the signatures found *in vivo* (Fig. 2C–K), we first tested the effect of γ-Tocotrienol on lactation organoids derived from BL/6^NZB^ females. We confirmed that induction of the cell cycle markers is only observed after addition of prolactin (Fig. 6A). Treatment with γ-Tocotrienol however, abolished the expression of cell cycle markers (Fig. 6A) and led to the expression of CHOP and p53 as well as cleavage of caspase 3 (Fig. 6A). Since this result suggests that γ-Tocotrienol reverses the pro-tumorigenic signature observed in BL/6^NZB^ females during lactation, we next tested if this also holds true *in vivo*. Since the inactivation of ER-stress signaling occurs sometimes in between day 14 and day 21 of lactation in BL/6^NZB^ females in order to mimic sustained activation of ER-stress, were initiated treatment with γ-Tocotrienol on day 10 of lactation by daily gavage for four weeks (Fig. 6B). First, treated and untreated females were analyzed for 45 days post-partum hyperplasia. We found that treatment with γ-Tocotrienol reduces the number (Fig. 6C) and size (Fig. 6D) of the mammary ducts. Further, analysis of the entire mammary gland by whole mount confirmed a drastic reduction in proliferation of the mammary glands upon treatment with γ-Tocotrienol (Fig. 6E), which was confirmed using staining with Ki67 (Fig. 6F).

To test the effect of γ-Tocotrienol on the growth of exogenous cancer cells, BL/6^NZB^ females were treated for four weeks by daily gavage starting at day 10 of lactation and EO771 cancer cells were injected in the mammary fat pad on day 17 of lactation (Fig. 6G) and tumor volumes were measured every 4 days. We found that γ-Tocotrienol significantly reduced tumor growth (Fig. 6H, I) and remarkably this effect was observed despite the fact that treatment with γ-Tocotrienol was completed prior to the injection of cancer cells.

We conclude that γ-Tocotrienol can reverse not only the pro-tumorigenic signature observed in BL/6^NZB^ females during lactation but also the post-partum hyperplasia and tumor formation *in vivo*.

## Discussion

Lactation is an ancient reproductive feature that pre-dates the origin of mammals. It originates from apocrine-like gland associated with hair follicles to provide moisture, nutrients and antimicrobials to shelled eggs. The evolution of placenta-based reproduction displaced the function of milk as a source of water and nutrients to eggs, to the secretion of complex milk. The nutrition provided by mammary gland-derived milk is essential to the survival of off springs across the mammalian kingdom. In humans, extensive studies have been performed to dissect the benefits of breast milk to newborns, but the benefits of breastfeeding to the mothers have received much less attention. Yet, a meta-analysis combining the results of 47 epidemiological studies across 30 countries and involving over 50, 000 women without breast cancer and nearly 100, 000 women with breast cancer led to the conclusion that the longer a woman breastfeed, the more protected she is against breast cancer^13^. However, the very large sample size required to reach this conclusion indicates that breastfeeding is not protective for all women. This observation raises two fundamental questions; first, what are the differences between women that benefit from the protective effect of lactation and those who do not and second, what is the mechanism of protection against breast cancer? Our study proposes answers to both questions.

Firstly, our study reveals a remarkable level of idiosyncrasy of lactation at all levels; organelles-network reorganization, global transcriptional dynamic in term of total number of genes over time, cell-type specific transcriptome and signaling pathways activated or repressed during lactation between the two genotypes. This unexpected idiosyncrasy however does not impact the nutritional function of lactation since females from both genotypes produce milk and successfully provide nutrients to their off springs, as no difference in the weight of pups is observed. Since the only difference between genotypes is the mitochondria, these observations indicate that the impact of mitochondrial haplotypes extends well beyond the mitochondrial itself and affects the communication with the ER and transcription in the nucleus. Lactation appears to be a stunning example of a physiological setting where this inter-organelle triad of communication alters the underlying biology. Our study raises the possibility that unlike the widely accepted notion that lactation is a uniform biological process between women, there may be multiple routes of lactation that vary between women based on their mitochondrial haplotypes.

Second, we show that the divergence of signaling pathways induced or repressed during lactation between genotypes result in anti-or pro-tumorigenic environment and that pro-tumorigenic lactation shares the genetic signature that was identified in post-partum breast cancers in humans^26^. Our results support the notion that difference in ER and mitochondrial stress responses during lactation is the mechanism of these divergent outcomes by affecting transcription via the large number of transcription factors involved in these stress responses. The impact of the response to ER-stress is supported by the observation that treatment with g-tocotrienol, a dietary supplement known to induce a pro-apoptotic ER-stress^40–42^ was able to reverse the outcome of lactation ex-vivo and *in vivo* in BL/6^NZB^ females.

Among the transcription factors, p53 is of particular interest. A large number of studies has now linked the regulation of ER-stress and metabolism to p53^27,29–33^. The mechanistic details of how these pathways affect p53 under normal physiological conditions remain to be characterized and our results offer an additional example. Our hypothesis is that the loss of p53 expression during lactation in BL/6^NZB^ females results from the difference in ER-stress and inadvertently allows the expansion of the PPBC-like sub-population of cells, which are characterized by the elevated cell cycle score and expression of the PPBC-regulon. Therefore, despite the fact that a small number of PPBC-like cells are detected in virgin mammary glands in both genotypes and also in lactating BL/6^C57^ females, our data suggest that it is the specific loss of p53 expression during lactation in BL/6^NZB^ females that allows their expansion. We postulate that these cells are primed for transformation such that upon acquisition of a sporadic oncogenic mutation, they lead to the formation of PPBC, therefore explaining their genetic overlap with the PPBC signature in humans. However, given that the induction of the ER-stress has been described in multiple cell types and is known to impact the biology of several immune cells^43–45^, it is likely that multiple cell types contribute to the outcome of lactation *in vivo*.

**Figure 7.**
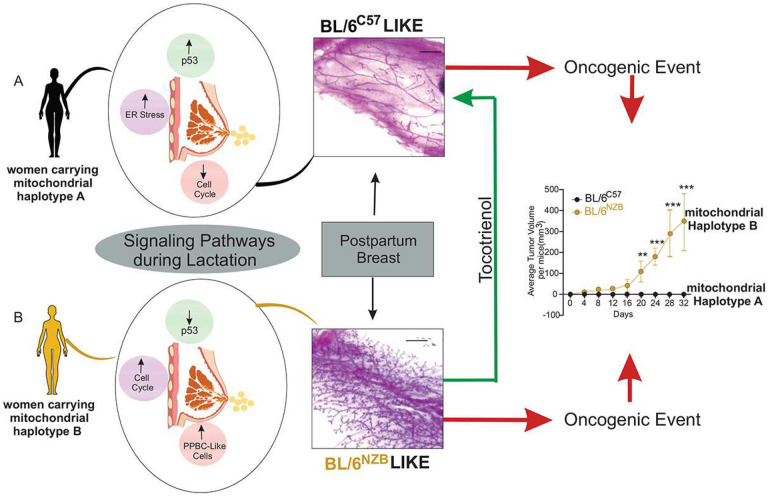
Schematic summary. A) in a hypothetical woman carrying a subset of mitochondrial haplotypes, indicated as mitochondrial haplotype A, lactation promotes a transcriptional and cellular landscape that is anti-tumorigenic and characterized by a downregulation of cell cycle genes and maintenance of p53 function, providing a protective effect against proliferation. In the event of an acquired oncogenic mutation, this environment protects against the formation of PPBC. B) in a hypothetical woman carrying another sub-set of mitochondrial haplotypes, indicated as mitochondrial haplotype B, lactation inadvertently results in loss of p53, increased expression of cell cycle genes allowing the formation of a pro-tumorigenic environment and the expansion of the PPBC-like sub-population of cells. In the event of an acquired oncogenic mutation, these PPBC-like cells lead to the formation of PPBC (Fig. 7B).

Since our mouse models recapitulate the natural diversity in mitochondrial genetics observed in the human population, the global implication of our findings is that for women carrying a subset of mitochondrial haplotypes, lactation promotes a transcriptional and cellular landscape that is anti-tumorigenic and provides the observed protective effect of lactation (Fig. 7A). However, in women carrying another sub-set of mitochondrial haplotypes, lactation inadvertently results in a pro-tumorigenic environment by allowing the expansion of the PPBC-like sub-population of cells, which are prone to transformation (Fig. 7B). Additionally, since we found that the growth of exogenously injected cancer cells from an entirely different genetic makeup, is also stimulated during lactation in BL/6^NZB^ females, our data indicate that other cell types, beyond the PPBC-like sub-population, also contribute to the pro-tumorigenic environment in these females.

In conclusion, our study revealed an unsuspected idiosyncrasy of lactation that is dictated by mitochondrial haplotypes. While the PPBC-like sub-population is detected in virgin females of both genotypes and BL/6^C57^ females during lactation, this idiosyncrasy results in the inadvertent inhibition of p53 specifically in the BL/6^NZB^ females resulting in their expansion and susceptibility to PPBC. Our findings offer a potential dietary intervention to reverse the pro-tumorigenic response to lactation into an anti-tumorigenic response therefore, holding the potential to enhance the beneficial effect of lactation to more women and reduce the incidence of PPBC.

## METHODS:

### Animals

All animal experiments were performed in accordance with IUCAC approved protocol. BL6/C57^NZB^ mice were originally generated and provided by our collaborator Dr. J.A Enriquez^25^. The BL6/C57^C57^ (C57BL/6JOlaHsd) strain was purchased from ENVIGO, Netherlands^25^. Female mice aged between 2 months and 6 months were put into breeding. After their 1^st^ pregnancy, mammary glands were collected from lactating female mice at 8, 14 and 21 days of lactation. For the analysis of post-partum samples, mammary glands were collected from mice after 45 days post weaning (21 days). Inguinal mammary glands were frozen on dry ice and kept for RNA seq (Bulk and Single Cell) and western Blot analysis. Thoracic mammary glands were collected for histology. E0771 murine mammary cancer cell line originally isolated from spontaneous mammary tumor in BL6/C57 mice was used to generate tumors in mammary glands in the mice following an earlier published protocol with slight modifications^46^. 1×10^5^ E0771 cells suspended in 25 μl Hank’s balanced salt solution (HBSS) and 25 μl growth factor reduced matrigel (GIBCO) was injected in both the inguinal mammary glands of lactating mice on 17^th^ day. Tumors volumes were measured 3 times per week using electronic callipers. The greatest longitudinal diameter (length) and the greatest transverse diameter (width) were measured, and tumor volumes were calculated by the modified ellipsoidal formula: volume=1/2(length×width2). For some experiments, the mouse was given olive oil (Vehicle) or δ-Tocotrienol (200 mg/kg in olive oil, twice daily) by oral gavage, 5 days/week from the 10^th^ day of lactation for 4 weeks^47^. All mice were housed in vivarium at Mount Sinai with ad libitium access to food and water.

### Whole Mounts

Inguinal mammary glands were removed from mice and partially airdried on standard microscope slides (Fisher Cat# 12–544–3) for 5–7 min. Then the glands were fixed overnight in 75% ETOH. 25% Glacial Acetic Acid. The slides with the glands were washed in 70% ethanol for 15 min before gradually changing to water. The glands were then stained with Carmine solution (1 g carmine (Sigma C1022) and 2.5 g aluminum potassium sulfate (SigmaA7167) in 500 ml dH2O) overnight followed by washing with MilliQ filtered water and dehydrated in an alcohol series. Finally, glands were immersed in xylene to clear them and then mounted in Permount (Fisher Cat # SP15–100).

### Histology and Immunohistochemistry

Tissues were fixed in 10% formalin (Thermo Fisher Scientific) for 24 h and then processed and embedded into paraffin for sectioning by the Biorepository Core Facility at Mount Sinai. Unstained paraffin embedded slides were obtained from the core facility. The slides were stained with Hematoxylin and Eosin. Images were captured using a Zeiss AX10 microscope. For immunohistochemistry, sections were deparaffinized in xylene twice for 5 min each. Then they were rehydrated through a decreasing ethanol series (100%, 90%, 70%) gradually changing to water. Antigen retrieval was done for 30 min at 90–100°C in 10 mM Sodium citrate buffer pH 6. The slides were slowly cooled down to room temperature, then t rinsed twice in Tris buffered saline (TBS) followed by antibody staining according to the manufacturer’s suggested protocol for the ImmPRESS Excel Amplified Polymer Kit (Vectorlabs Cat# MP-7601). Ki67 were detected with LS-C141899–0.1 from LifeSpan Biosciences diluted 1:100 for 1 h at room temperature in a humidified chamber.

### Western Blotting

Flash frozen mammary glands were pulverized and lysed in cold NP-40 lysis buffer with protease inhibitors (50 mM Tris, pH 7.5, 250 mM NaCl, 5 mM EDTA, 0.5% Nonidet P-40, 50 mM NaF, 0.2 mM Na3 VO4, 1 g/ml leupeptin, 1 g/ml pepstatin, 100 g/ml phenylmethylsulfonyl fluoride) for 10 min, sonicated for 5 s at 20% amplitude and then centrifuged at 14,000 rpm for 20 minutes at 4°C and then the supernatant was collected. Protein concentrations were measured using the Bradford method (Bio-Rad Protein Assay). Equal amounts of protein were loaded and separated by SDS-PAGE electrophoresis and then transferred to nitrocellulose membrane (GE Healthcare) followed by 30 min blocking. The membranes were then probed with the following primary antibodies overnight at 4°C: HSP60 (BD Transduction 611563), ATF5 (abcam ab184923), ATF4 (Proteintech 60035–1-Ig), ClpP (abcam ab124822), CHOP (Cell Signaling 2895S), BIP (BD Transduction 610978), PERK (Cell Signaling Technology C33E10), pPERK (Thermo Scientific MA515033), PARP-CL (Cell Signaling Technology 9546S), Caspase 3-CL (Cell Signaling Technology 9661), XBP1s (Santa Cruz Technology sc-7160), CDK2 (Santa Cruz Technology sc-163), CDK4 (Santa Cruz Technology sc-260), cyclin D1 (Santa Cruz Technology sc-718), cyclin E (Santa Cruz Technology sc-481) and Actin (EMB Millipore MAB1501R). Blots were then washed with TBST (Tris Buffered Saline with 0.1% Tween 20, pH 7.4) and probed with horseradish peroxidase conjugated anti-mouse (Jackson ImmunoResearch or KwikQuant) or anti-rabbit secondary antibodies (Thermo Fisher Scientific or KwikQuant) for 2 h. This was followed by 3–4 washes with TBST and the protein bands were detected using enhanced chemiluminescence (GE Healthcare or KwikQuant).

### Isolation of primary mammary epithelial Organoids

Primary mammary epithelial organoids were isolated from 3 months old non lactating female mice. Thoracic and inguinal mammary glands were collected, and then visible lymph nodes were excised. The mammary glands were finely chopped to approximately 1-mm3 pieces and digested in Dulbecco’s in modified Eagle medium (DMEM)/F12 (Thermo Fisher Scientific 10-565-042) containing 0.2% trypsin (Thermofisher Scientific 27250018), 0.2% Collagenase (Thermo Fisher Scientific 17104019), 5% FCS and 1000 U/ml Pennicillin/Streptomycin for 30 min at 37°C with shaking at 100 rpm. Immediately after digestion, digested mammary glands were centrifuged at 200g for 10 min. The pellets were resuspended in DMEM/F12 media and treated with 2U/μl DNase for 5 min with gentle shaking at room temperature, followed by centrifugation at 200g for 10min. The pellets were resuspended in fresh DMEM/F12 media and subjected to 3 rounds of deferential centrifugation at 200g for 10 sec to enriches the organoids. The number of organoids is manually counted under the microscope.

### 3D organoid culture and imaging

Mammary organoids were freshly isolated from non-lactating 3 months old female mice and mixed with growth factor reduced Matrigel (Corning, United States) and plated in domes in 96-well culture plate (one dome per well, 80 μL of Matrigel per dome). 200 to 500 organoids per dome were seeded for experiments. Then the Matrigel was allowed to set for 45–60 min at 37°C. After that the 3D organoid cultures were overlaid with MEC media [DMEM/F-12 media (Thermo Fisher Scientific 10-565-042) containing 10 % FBS, 1% P/S and 1% Insulin-Transferin-Selenium (Corning 25–800-CR)] and incubated at 37°C in a humidified atmosphere with 5% CO2 for 24 h. After 24 h the MEC media is replaced with BOM Media [MEC media supplemented with different growth factors: 2.5 nM FGF-basic (Peprotech 450–33), 2.5 nM FGF7 (Peprotech 450–60), 2.5 nM FGF10 (Peprotech 450–61), 50 ng/mL EGF (Peprotech 315–09)]. The organoids are cultured in BOM Media for 6 days and media was changed every 2 days. On the 6^th^ Day, lactation was induced by replacing the BOM media with lactation media [BOM media supplemented with 1 μg/mL prolactin (R&D Systems 682-PL-050), 1 μg/ml hydrocortisone (Sigma-Aldrich H0888–5G)]. To induce contraction in the organoids 40 μg/ml recombinant oxytocin (Sigma-Aldrich 06379–1MG) was added. For some experiments, the organoids were treated 20 μg/ml of δ-Tocotrienol (Sigma-Aldrich T0577–10MG) from the fourth day. Live imaging was captured with a Zeiss AxioObserver equipped with a fully enclosed incubation system to provide constant temperature at 37ᵒC with 5% CO2. The image capture interval was either 15 or 20 minutes for durations of ~24hrs. Movies were analyzed frame by frame. For western blot analysis, each well is washed with PBS for 3 times. Then the organoids are dissolved in warm SDS loading buffer and boiled for 5 min and subjected to SDS PAGE. Immunofluorescence staining was performed as previously described (Jenkins et al 2012, 2014). Briefly, 3D organoid cultures were fixed with 4% paraformaldehyde before being washed 2 times in PBS 5 minutes each and permeabilized with 0.5% tween 20. Staining for pRB in blocking buffer (10% horse serum, 2% bovine serum albumin, and 0.5% Triton X-100 in PBS) for 1 hour gently shaking at room temperature. Primary antibody (Cytokeratin 14–0.5ml (Lieca Biosystems NCL-L-LL002) or p-RB (Cell Signaling S780) was suspended in blocking buffer diluted 1:1 with PBS for 3hours before washing and detection with either AlexFlour 488 or AlexaFlour 594 conjugated secondary antibodies (1:100) for 1 hour. Actin was stained with Phalloidin-iFluor 594 Reagent (ab176757) according to the manufacturer’s specifications. Organoids were then counterstained with DAPI and moved to slide for imaging.

### Bulk RNA Sequencing & Analysis

Inguinal mammary glands from mice were flash frozen and sent to GENEWIZ, LLC, NJ, USA for analysis. Each sample was analyzed 3 times. 3 mice per group were analyzed. The RNA seq data was submitted to Biojupies and Enrichr for further analysis.

### Single Cell RNA Sequencing and Analysis

Freshly isolated snap frozen Inguinal mammary glands were submitted to GENEWIZ, LLC, NJ, USA for nuclei isolation and GEM (Gel Bead-based Emulsion) single cell RNA seq analysis on the 10x Genomics platform.Analysis was performed using the Cellranger (10xGenomics) count pipeline. Cell populations were identified using the Loupe Browser (v8.0.0, 10x Genomics) by the following features: Mfge8, Trf, Csn3, Wfdc18, Ltf (Luminal Alveolar (AV) cells), Prlr, Pgr, Esr1, Cited1, Prom1 (Luminal Hormone Sensitive (HS) cells), Krt17, Krt14, and Krt5 (Myoepithelial cells). Proliferating cells were identified by the combined feature sum expression of Cenpe, Ccna2, Ccnb2, Mcm6, Ccnf, and Bud1 as described^48^. Regulon expressing cells were identified by the combined feature sum expression of Bclaf1, Cux1, E2f1, E2f4, Esr1, Foxm1, Gtf2b, Max, Myc, Nfya, Nr4a1, Nrf1, Smarca4, and Taf1 as described^26^. The PPBC-like sub-population was identified among total barcodes by co-expression of both the Proliferating Cell and Regulon feature list. A filter cutoff to identify this population was set for half of the maximum Log2 feature sum for both feature lists. Differential gene expression for the Luminal-HS population was performed using the Loupe browser (v8.0.0) for each sample and assembled into a single .csv file that was uploaded to the Biojupies platform(https://doi.org/10.1016/j.cels.2018.10.007) to produce hierarchically clustered heatmaps using clustergrammer (developed by the Ma’ayan Laboratory at Mt. Sinai). The top 40 most differentially expressed genes between Bl/6 C57 and Bl/6 NZB LacD21 samples were then entered in EnrichR^49,50^ (https://doi.org/10.1002/cpz1.90) for pathway analysis across multiple databases.

### Quantification and Statistical Analysis

Statistical significance was defined as a p value below 0.05. ns = not significant, * p < 0.05, ** p < 0.01, *** p < 0.001, **** p < 0.0001. Data displayed as mean ± standard deviation (SD). Statistical analyses were performed using GraphPad Prism Software 8. Statistical significance was determined using Studentś t-test. Immunofluorescent images were processed uniformly, and Immunofluorescence signal was quantified using ImageJ to measure area positive for fluorescent signal.

## Data Availability

All sequencing data is currently being processed for submission to public repository and will be made available to reviewers.
